# College and Hospital Notes

**Published:** 1900-10

**Authors:** 


### COLLEGE AND HOSPITAL NOTES.

THE Medical Department of the University of Buffalo opened its fifty-fifth annual course of instruction at its building on High Street, Monday evening, September 24, 1900. Dr. Eugene A. Smith, adjunct professor of surgery, delivered the inaugural address to a large audience of students and citizens, which will be printed in the next issue of the JOURNAL. The list of matriculates is a large one and the college is in a prosperous condition. The University is about to build an annex and enlarge its scope. A three-story building is to be reared in the immediate vicinity of the General Hospital, with an endowment fund of $40,000 that has become available during the last year.

DR. JOHN B. MURPHY, of Chicago, has accepted a professorship in surgery and clinical surgery in the Northwestern University Medical School—Chicago Medical College. Dr. Murphy has been appointed surgeon-in-chief of Mercy Hospital with the direction of the surgical teaching in that hospital. He will give two clinics each week at the hospital. The hospital now contains 260 beds with abundance of clinical material. A new amphitheatre with a seating capacity of 300 is in progress of construction.

Dr. Archibald Church has been recently appointed professor of nervous and mental diseases in Northwestern University Medical School—Chicago Medical College—and head of the neurological department.

